# Determinants of Patient Delay in Seeking Diagnosis and Treatment among Moroccan Women with Cervical Cancer

**DOI:** 10.1155/2016/4840762

**Published:** 2016-11-02

**Authors:** Fatima Ouasmani, Zaki Hanchi, Bouchra Haddou Rahou, Rachid Bekkali, Samir Ahid, Abdelhalem Mesfioui

**Affiliations:** ^1^Laboratory of Genetic, Neuroendocrinology and Biotechnology, Faculty of Sciences, Ibn Tofail University, Kenitra, Morocco; ^2^National Institute of Oncology, Rabat, Morocco; ^3^Lalla Salma Foundation-Prevention and Treatment of Cancers, Rabat, Morocco; ^4^Laboratory of Epidemiology and Clinical Research, Medical and Pharmacy School, Mohammed V University, Rabat, Morocco

## Abstract

*Introduction*. This study sought to investigate potential determinants of patient delay among Moroccan women with cervical cancer.* Methods*. A cross-sectional study was conducted from June 2014 to June 2015 at the National Institute of Oncology in Rabat. Data were collected using questionnaire among patients with cervical cancer locally advanced or metastatic (stages IIA–IVB). Medical records were abstracted to complete clinical information. An interval longer than 90 days between discovery of initial symptoms and presentation to a provider was defined as a patient delay.* Results*. Four hundred and one patients with cervical cancer enrolled in this study. The mean age was 52.4 years (SD = 11.5). 53.6% were illiterate. Abnormal vaginal bleeding was identified for 65.8% of patients. 60.1% were diagnosed at stages IIA-IIB. 55.4% were found having patient delay. The regression analyses showed the association between literacy (*p* < 0.001), distance of the place of the first consultation (*p* = 0.031), abnormal vaginal bleeding as an earlier symptom (*p* < 0.001), stage at diagnosis (*p* < 0.03), knowledge of symptoms (*p* < 0.001), knowledge of causes (*p* = 0.008), and practice of gynecological exam during the last three years (*p* = 0.018) and the patient delay.* Conclusion*. Educational messages should aim at increasing awareness of cervical cancer, assisting women in symptom recognition, and encouraging earlier presentation.

## 1. Introduction

Cervical cancer with an estimated 528,000 new cases is the fourth most common cancer in women worldwide. The large majority, around 85% of the global burden of the disease, occurs in the less developed regions [1]. In developed countries, the incidence of cervical cancer has decreased dramatically since the introduction of cervical screening programs. However, in developing countries the disease incidence remains high. Cervical cancer is the most curable form of any human cancer if detected early at the precancerous stage [2, 3] but the challenge is that 80% of women in the developing countries seek medical care after they have developed signs and symptoms [4]. One of the most important prognostic factors for cervical cancer is how early the disease is when detected and how far it has spread. Delay in diagnosis and treatment continues to be the greatest hurdle to be overcome in the fight to cure cancer [5, 6]

In Morocco, cervical cancer is increasingly becoming a major public health concern. It is considered the second leading cancer in women and causes high morbidity and mortality. The majority of cases present to hospitals at late stages when treatment is less feasible [7]. Nearly 2258 cases of cervical cancer are diagnosed annually and 1076 die, with an age-standardized incidence rate of 14.3 per 100,000 per annum, and it accounts for 12.8% of total female cancer [8]. Though the incidence rate of cervical cancer is low in Morocco, the mortality ratio is high which indicates that most of the cancer cases are diagnosed in advanced stages, 43.7% in stage II and 38.1% in stages III and IV [9]. Advanced cervical cancer is one of the major causes of cancer related mortality in women specially in low- and medium-income countries mostly due to poor access to appropriate management [3, 6]. Factors such as unavailability to routine screening, inadequate follow-up of abnormal pap smears, and possibly low awareness of women's population regarding the course of the disease could explain the higher mortality of cervical cancer [10].

Recently, the concept of delayed diagnosis has become an important issue in the cancer prevention and treatment. The concept is categorized in four components including patient delay, health care provider delay, referral delay, and system delay, though all of the mentioned delays have important role in the prevention, diagnosis, and management of the disease but it seems that in developing countries patient delay has more crucial role [11, 12].

Delays may occur at different stages of the cancer diagnostic journey and have been commonly defined as being either patient focused or health care provider focused [5, 12].

However, to our knowledge, little information is available concerning knowledge and practice as determinants of patient delay in seeking diagnosis and treatment among women with cervical cancer in Morocco. The present study was, therefore, attempted to address some extensive information directly from patients in* a hospital* based setting and sought to identify potential determinants of patient delay in Moroccan women with cervical cancer.

## 2. Methods

We conducted a cross-sectional study at the National Institute of Oncology Sidi Mohammed Ben Abdellah in Rabat, specifically in gynecological and breast pole. This institute, which is part of the Ibn Sina University Hospital of Rabat, is dedicated exclusively to fighting cancer. It is the referral hospital for cancer care where most of the cases are diagnosed and treated. The protocol of this study was approved by Ethics Committee for Biomedical Research, Faculty of Medicine and Pharmacy of Rabat, Mohammed V University. After obtaining written informed consent from each patient, the data was collected between June 2014 and June 2015 using a face to face structured questionnaire. This tool was pretested and modified before final data collection was done. Data on clinical variables was extracted from hospital record of the patients under study.* All interviews were conducted by the searcher*.

Moroccan women who had already started their treatment for cervical cancer, who have a hospital record in the National Institute of Oncology, who attend the hospital for processing or checking during the study period, and who have signed a letter of informed consent to participate in the study were included in the study. A consecutive series was carried out for eligible patients with cervical cancer locally advanced or metastatic (IIA–IVB) (The International Federation of Gynecology and Obstetrics (FIGO), Committee on Gynecologic Oncology) [13]. Patients diagnosed with cervical cancer but who have not yet started treatment and patients with psychiatric disorders were excluded from the study.

The sample size was calculated from a proportion of 54.5% cervical cancer patients who delayed seeking health care [9] with 5% of precision and 95% for confidence interval. The minimum sample size *n* = 373 was obtained using the formula developed by Schwartz [14]. All cervical cancer patients in one year of the study that meet the inclusion criteria were involved and were able to reach a total of 401.

The variables included in data analysis were age, marital status, education level, education status of husband, residence, occupation, socioeconomic status, health insurance, remoteness of the first consultation place, medical history, type of earlier symptoms, personal history of cancer, family history of cancer, histopathology, clinical stage of tumor, knowledge of cervical cancer symptoms, causes, screening program, transmission mode, and treatment, and practice of pap smear, gynecological examination, and screening program.

Patient delay refers to a prolonged interval between discovery of initial symptoms and presentation to a provider* who can be a general practitioner, a gynecologist, a midwife, or a nurse* and is typically defined as greater than 90 days (three months) [15].

The patients were divided into two groups, those who sought medical care within 90 days or less and those who sought medical care more than 90 days after the first symptom recognition. Patients were excluded if this time interval could not be confirmed.

Data analysis was performed in computer using the software Statistical Package for Social Sciences (SPSS) version 13. Chi Square test and *p* value at 95% confidence level were used to predict the association. Multivariate binary logistic regression analysis was performed to measure the association between study variables and magnitude of patient delay. Adjusted odds ratio (OR) with its corresponding 95% confidence interval (CI) was calculated. Significance was when *p* < 0.05.

## 3. Results

The distribution of the patients according sociodemographic and clinical data characteristics is presented in Table 1. In total of four hundred and one patients enrolled in the study, 53.4% were older than 50 years. The mean age was 52.4 years (SD = 11.48) with the range from 28 to 83 years. 53.6% were illiterate and 63.3% were married. More than half of patients were urban inhabitants (68.6%). In 78.6% of the patients, the first consultation place from the residence was at a distance of less than 3 km; of all patients 81% were poor and 80.8% were unemployed. The majority of patients had a social security (97.5%).

As an earlier symptom, abnormal vaginal bleeding was identified for 65.8% of patients. Greater proportion, 87%, of the patients had squamous cell tumor type and 60.1% of total patients were diagnosed at stages IIA-IIB. Only 10.2% of patients had a family history of cancer.

Regarding knowledge and practice data, the results showed that 48.9% of all patients have never heard about cervical cancer and 60.3% did not know the abnormal vaginal bleeding as cervical cancer symptoms. Greater proportion (90.3%) did not have any idea about causes of cervical cancer. 51.6% of cases have never heard about screening program and 74.1% did not know transmission mode. More than half (58.9%) of patients have never done a pap smear test and 55.9% did not have a gynecologic examination during last three years and 82% have never done a screening test (Table 2).

The association between sociodemographic characteristics and patient delay is presented in Table 3. A wide variety of variables were associated univariately with patient delay such as age (adjusted OR = 0.509, CI: 0.341–0.759, *p* < 0.001) and level of education as protector factors, respectively, for primary and secondary and high level (adjusted OR = 0.430, CI: 0.259–0.713, *p* < 0.001, adjusted OR = 0.071, CI: 0.038–0.133, *p* < 0.001). The secondary and higher level of husband education was also found as a protector factor (adjusted OR = 0.176, CI: 0.085–0.365, *p* < 0.001). Rural residence was considered as a risk factor (adjusted OR = 1.888, CI: 1.219–2.925, *p* = 0.004). Occupation was considered as a protector factor (adjusted OR = 0.439, CI: 0.264–0.730, *p* = 0.002). Low socioeconomic status was found as a risk factor for patient delay (adjusted OR = 3.927, CI: 2.280–6.765, *p* < 0.001) and remoteness more than 6 km for the first consultation was found as a risk factor (*p* < 0.05).

After multivariate analysis, only literacy status of women and remoteness of place of first consultation were found significantly associated with patient delay. Lower risk of patient delay was observed for women who were literate (*p* < 0.001) but high risk was observed for patients who were more than 10 km far from the first consultation (adjusted OR = 1.68, CI: 1.08–2.60, *p* = 0.02).

The association between clinical variables and patient delay is presented in Table 4. All variables which were statically significant on univariate analyses at *p* (0.20 levels) were included in multivariate analysis. Abnormal vaginal bleeding as an earlier symptom was found as a protector factor (adjusted OR = 0.345, CI: 0.218–0.548, *p* < 0.001). Stages III and IV at diagnosis were found as a risk factor for patient delay (adjusted OR = 2.113, CI: 1.348–3.314, *p* < 0.001, adjusted OR = 11.439, CI: 1.431–91.443, *p* = 0.022).

The association between knowledge and practice in cervical cancer and patient delay is summarized in Table 5. Bivariate analysis showed that patients with no knowledge of cervical cancer (*p* < 0.0001), symptoms (*p* < 0.0001), causes (*p* < 0.0001), screening program (*p* < 0.0001), transmission mode (*p* = 0.0001), and treatment (*p* < 0.0001) were more likely to delay.

Furthermore, patients who have already made a pap smear (*p* < 0.0001) were less likely to delay. But patients who did not practice a gynecological examination during the last 3 years (*p* < 0.0001) and screening test (*p* < 0.0001) were more likely to delay.

The multivariate comparison of those factors showed that knowledge of abnormal vaginal bleeding as a symptom was found as a protective factor (adjusted OR = 0.069, CI: 0.020–0.231, *p* < 0.001) compared to the vaginal discharge and pain. Knowledge of causes was found also as a protective factor (adjusted OR = 0.094, CI: 0.016–0.546, *p* = 0.008). Women were more likely to delay if they did not practice a gynecological examination during the last 3 years (adjusted OR = 4.517, CI: 1.295–15.762, *p* = 0.018).

## 4. Discussion

This paper is the first that studied knowledge and practice as determinants of patient delay in women with cervical cancer in Morocco. Four hundred and one (401) patients with cervical cancer enrolled in this study. Two hundred and twenty-two (55.4%) were found having patient delay. The rate indicated the necessity of general screening program or improvement of population awareness in this regard.

The present findings are consistent with a research conducted in Morocco showing that sixty percent (60%) of women had a patient delay [9].

Among the sociodemographic characteristics of patients, education status of women was found significantly associated with patient delay. Lower risk of patient delay was observed for women who were literate. This result is in agreement with some studies [9, 16, 17], but in contrast with others [18, 19].

Our study did not demonstrate association between age, health insurance, place of residence, and patient delay. A study conducted in Sudan [20] has shown that older age is a predictor for patient delay. This study investigated also the association between rural residence, not having insurance, and delay at presentation of cervical cancer.

The findings showed that remoteness of place of the first consultation was found significantly associated with patient delay. High risk was observed for patients who were 3 km and more far from the first consultation. Our results concur closely with the available data in the literature [9, 11, 21].

While our findings are consistent with some studies, they were in contrast with others. A study conducted in China indicated that being unmarried was the high risk factor for delayed reporting of cervical cancer [22].

Of the 401 patients studied, 160 (39.9%) presented with stage III or IV disease of which 110 have presented a patient delay. This could be due to excessive delay that allowed the progression of the disease to advanced stage. The advanced stage at presentation might be due to the fact that most cancer in low- and middle-income countries is detected at later stages. There was a significant association between patient delay and late stage at presentation. The present study is consistent with a previous study conducted in Uganda by Galukande et al. that show that patient delay is more prevalent in patients with advanced stage at presentation [23].

This study revealed that women having abnormal vaginal bleeding such as postcoital bleeding, intermenstrual bleeding, or postmenopausal bleeding as early symptom were less likely to have patient delay. This can be explained by the fact that gynecological bleeding is usually perceived as more urgent than gynecological infection or pelvic pain. Women usually tend to ignore the mild gynecological symptoms such as vaginal discharge considering it as a general problem until it becomes warning symptoms such as vaginal bleeding [24].

Patients were typically less likely to delay if they experienced a more serious symptom, such an alarming symptom like bleeding. These findings are further corroborated with a study conducted in Morocco, where increased risks for patient delay were observed in women who did not have vaginal bleeding as the first symptom [9].

The patients who lacked knowledge on cervical cancer and its symptoms were at risk for patient delay. The low levels of symptom awareness may partly explain why the type of symptom is consistent with risk factors for delayed patient presentation [25]. Patients may fail to recognize or appreciate atypical or vague symptoms, which may mediate delayed presentation. A systematic review that examined the risk factors for delayed presentation in cancer found that nonrecognition of symptom type and infrequent care-seeking were the predominant risk factors for delayed presentation for gynecological cancers [23]. Many other studies have identified knowledge, availability, or lack, thereof, as a factor influencing access to health care services [26, 27].

In the current study, greater patient delay risk was found among patients who did not practice a gynecological examination during last 3 years. There was evidence that regular visits to medical practitioners, including attendance for routine screening, were associated with shorter delay in patients with gynecological cancers [28]. A study conducted in Iran by Behnamfar and Azadehrah found that history of pap smear had significant association with delay at presentation [29], even though our findings did not demonstrate this association.

The respondents showed a somewhat poor knowledge of causes of cervical cancer with 90.3% respondents admitting they do not know what causes the disease; some even after having the disease for several years they still did not know about the HPV virus. The others believed that cervical cancer was due to a variety of factors even though a few patients knew it was sexually transmitted. Similar findings were noted by other studies carried out in India and Maldives [30, 31].

In the present study, over half (55.4%) of the patients with cervical cancer were over 90 days to check for symptoms and reported patient delay. Such a high proportion may be explained by the fact that knowledge level can determine the kind of action one may take which could be either negative or positive. It is important not to lose sight of the fact that low knowledge about cervical cancer potentially precludes uptake of early presentation [32]

The determinants of patient delay can be different in different places. One explanation for such a difference might relate to the socioeconomic context where patients live.

However, due to the amalgamation of many factors that lead to patient's delay, prevention interventions should focus on targeted women to promote early presentation. Although in Morocco a nationwide program for early detection of cervical cancer was implemented in 2010 using visual inspection with acetic acid (VIA) as the screening tool several challenges to national scale-up were detected such as low compliance with the screening program. A national awareness campaign and mass communication are required to improve screening participation [33].

Further research is needed to clarify relevant other risk factors for delayed time to presentation for some particularly underresearched cancers, such as cervical cancer.

This study was not free of limitations. It is a cross-sectional design and constituted of patients attending a tertiary hospital in the country capital and hence it might not be representative of all the Moroccan patients affected by cervical cancer. Also, the duration of symptoms which were described by the patients might be biased.

## 5. Conclusion

This study sought determinants of the patient delay in women with cervical cancer. The findings point to poor knowledge among most Moroccan patients on the actual nature of cervical cancer, its symptoms, and causes. Most of the women did not know about cervical cancer prior to their diagnosis. Knowledge of cervical cancer and practice of examination are important elements in determining whether women will take preventive measures against the disease. Educational messages to the general population should aim at increasing awareness of cervical cancer, assisting women in symptom recognition, and encouraging earlier presentation. It seems that in order to achieve more conclusive results further studies with prospective design are needed.

## Figures and Tables

**Table 1:** Sociodemographic and clinical characteristics of the study population (*n* = 401).

Characteristics	*n*	%
*Age (year)*		
≤50	187	46.6
>50	214	53.4
*Marital status*		
Married	254	63.3
Single	9	2.3
Divorced	56	14
Widow	82	20.4
*Education status*		
Illiterate	215	53.6
Primary level	93	23.2
Secondary and higher level	93	23.2
*Education status of husband (n* = 254)		
Illiterate	54	13.5
Primary level	85	21.2
Secondary and higher level	115	28.7
*Residence*		
Urban	275	68.6
Rural	126	31.4
*Occupation*		
Employment	77	19.2
Unemployment	324	80.8
*Socioeconomic status*		
Low	325	81
Moderate	69	17.3
High	7	1.7
*Social security*		
Yes	391	97.5
No	10	2.5
*Distance to the first consultation (Km)*		
≤3 km	315	78.6
[3–6[	17	4.2
[6–10[	36	9.0
≥10	33	8.2
*Medical history*		
Yes	105	25
*Type of earlier symptoms*		
Abnormal vaginal bleeding (between periods, after sex, postmenopausal)		
Yes	264	65,8
Vaginal discharge		
Yes	69	17,2
Pain (painful sex, pelvic pain, dysuria)		
Yes	139	34,6
*Personal history of cancer*		
Yes	3	0.7
*Family history of cancer*		
Yes	41	10.2
*Histopathology*		
Squamous cell	349	87
Adenocarcinoma	52	13
*Stage at diagnosis*		
IIA-IIB	241	60.1
IIIA-IIIB	145	36.2
IVA-IVB	15	3.7
*Patient delay (days)*		
≤90	179	44.6
>90	222	55.4

**Table 2:** Description of knowledge and practice in cervical cancer (*n* = 401).

Characteristics	*n*	%
*knowledge of cervical cancer*		
Yes	205	51.1
*Knowledge of symptoms*		
Abnormal vaginal bleeding (between periods, after sex, postmenopausal)		
Yes	158	39.6
Vaginal discharge		
Yes	71	82.3
Pain (painful sex, pelvic pain, dysuria)		
Yes	102	25.7
*Knowledge of causes*		
Yes	39	9.7
*Knowledge of transmission mode*		
Yes	104	25.9
*Knowledge of screening program*		
Yes	194	48.4
*Knowledge of HPV vaccine*		
Yes	45	11.2
*Knowledge of treatment*		
Yes	267	66.5
*History of pap smear*		
Never	236	58.9
More than 3 years	105	26.1
During last 3 years	60	15
*Practice of gynecological examination during last 3 years*		
Yes	177	44.1
*Practice of screening test by visual inspection with acetic acid (VIA) *		
Yes	72	18

**Table 3:** Associations between delayed reporting demographic and socioeconomic variables.

Categorial variables	≤90 days (*n* = 179)	>90 days (*n* = 222)	Univariate analysis			Multivariate analysis		
	*n* (%)	*n* (%)	OR	95% CI	*p*	OR	95% CI	*p*
*Age (year)*								
≤50	100 (53.5)	87 (46.5)	0.509	[0.341–0.759]	0.001^**∗**^	0.802	[0.817–3.977]	0.145
>50	79 (36.9)	135 (63.1)						
*Marital status*								
Married	114 (44.9)	140 (55.1)						
Unmarried	65 (44.2)	82 (55.8)	1.027	[0.683–1.546]	0.897			
*Education status*								
Illiterate	58 (27)	157 (73)						
Primary level	43(46.2)	50 (53.8)	0.430	[0.259–0.713]	0.001^**∗**^	0.239	[0.099–0.574]	0.001^**∗∗**^
Secondary and higher level	78 (83.9)	15 (16.1)	0.071	[0.038–0.133]	0.001^**∗**^	0.026	[0.007–0.095]	0.001^**∗∗**^
*Education status of husband*								
Illiterate	13 (24.1)	41 (75.9)						
Primary level	27 (31.8)	58 (68.2)	0.681	[0.314–1.476]	0.330	0.845	[0.367–1.948]	0.693
Secondary and higher level	74 (64.3)	41 (35.7)	0.176	[0.085–0.365]	0.001^**∗**^	1.219	[0.421–3.529]	0.715
*Residence*								
Urban	136 (49.5)	139 (50.5)						
Rural	43 (43.1)	83 (65.9)	1.888	[1.219–2.925]	0.004^**∗**^	0.451	[0.178–1.146]	0.094
*Occupation*								
Employed	47 (61)	30 (39)						
Unemployed	132 (46.7)	192 (59.3)	0.439	[0.264–0.730]	0.002^**∗**^	1.528	[0.496–4.708]	0.461
*Socioeconomic status*								
Low	125 (38.5)	200 (61.5)	3.927	[2.280–6.765]	0.001^**∗**^	1.525	[0.606–3.841]	0.370
Moderate and high	54 (71.1)	22 (28.9)						
*Social security*								
Yes	173 (44.2)	218 (55.8)						
No	6 (60)	4 (40)	0.529	[0.147–1.904]	0.330			
*Distance to the first consultation (Km)*								
<3	155 (49.2)	160 (50.8)						
[3–6[	5 (29.4)	12 (70.6)	2.325	[0.800–6.754]	0.121^**∗**^	2.953	[0.262–33.248]	0.381
[6–10[	6 (25)	27 (75)	2.906	[1.324–6.378]	0.008^**∗**^	2.720	[0.758–9.770]	0.125
≥10	10 (30.3)	23 (69.7)	2.228	[1.027–4.834]	0.043^**∗**^	4.943	[1.162–21.035]	0.031^**∗∗**^

^*∗*^Model included variables that were significant on univariate analyses at *p* (0.20 level).

^*∗∗*^Significant at *p* value ≤ 0.05.

**Table 4:** Association between clinical factors and patient delay, identified by multivariate analysis.

Categorial variables	≤90 days (*n* = 179)	>90 days (*n* = 222)	Univariate analysis			Multivariate analysis		
	*n* (%)	*n* (%)	OR	95% CI	*p*	OR	95% CI	*p*
*Medical history*								
Yes	52 (50)	52 (50)						
No	127 (42.8)	170 (57.2)	1.339	[0.855–2.095]	0.202^**∗**^	1.332	[0.827–2.145]	0.238
*Earlier symptoms*								
Abnormal vaginal bleeding (between periods, after sex, postmenopausal)								
Yes	158 (46,3)	183 (53,7)	0.335	[0.214–0.523]	0.001^**∗**^	0.345	[0.218–0.548]	0.001^**∗∗**^
No	21 (35)	39 (65)						
Vaginal discharge								
Yes	31 (50)	31 (50)						
No	148 (43,7)	191 (56,3)	1.014	[0.602–1.708]	0.958			
Pain (painful sex, pelvic pain, dysuria)								
Yes	92 (39.8)	139 (60.2)						
No	87 (51.2)	83 (48.8)	0.631	[0.423–0.942]	0.024^**∗**^	1.255	[0.812–1.940]	0.307
*Personal history of cancer*								
Yes	2 (66.7)	1 (33.3)						
No	177 (44.5)	221 (55.5)	2.497	[0.225–27.760]	0.456			
*Family history of cancer*								
Yes	23 (56.1)	18 (43.9)						
No	156 (43.3)	204 (56.7)	1.671	[0.871–3.204]	0.122^**∗**^	1.630	[0.812–3.272]	0.170
*Histopathology*								
Squamous cell	157 (45)	192 (55)						
Adenocarcinoma	22 (42.3)	30 (57.7)	1.115	[0.619–2.010]	0.717			
*Stage at diagnosis*								
IIA-IIB	129 (53.5)	112 (46.5)						
IIIA-IIIB	49 (33.8)	96 (66.2)	2.257	[1.472–3.459]	0.001^**∗**^	2.113	[1.348–3.314]	0.001^**∗∗**^
IVA-IVB	1 (6.7)	14 (93.3)	16.121	[2.087–124.507]	0.008^**∗**^	11.439	[1.431–91.443]	0.022^**∗∗**^

^**∗**^Model included variables that were significant on univariate analyses at *p* (0.20 level).

^**∗∗**^Significant at *p* value ≤ 0.05.

**Table 5:** Association between knowledge and practice and patient delay identified by multivariate analysis.

Categorial variables	≤90 (*n* = 179)	>90 (*n* = 222)	Univariate analysis			Multivariate analysis		
	*n* (%)	*n* (%)	OR	95% CI	*p*	OR	95% CI	*p*
*Previously heard about cervical cancer*								
Yes	155 (75.6)	50 (24.4)						
No	24 (12.2)	172 (87.8)	22.217	[13.039–37.855]	0.001^**∗**^	2.707	[0.834–8.753]	0.098
*Knowledge of symptoms*								
Abnormal vaginal bleeding (between periods, after sex, postmenopausal)								
Yes	141 (89.8)	16 (10.2)	0.020	[0.011–0.038]	0.001^**∗**^	0.069	[0.020–0.231]	0.001^**∗∗**^
No	37 (15.2)	206 (84.4)						
Vaginal discharge								
Yes	60 (84.5)	11 (15.5)						
No	119 (36.1)	211 (63.9)	9.969	[4.894–19.102]	0.001^**∗**^	0.874	[0.284–2.692]	0.814
Pain (painful sex, pelvic pain, dysuria)								
Yes	93 (89.4)	11 (10.6)						
No	86 (29)	211 (71)	20.743	[10.579–40.673]	0.001^**∗**^	1.956	[0.483–7.926]	0.347
*Knowledge of causes*								
Yes	40 (95.2)	2 (4.8)	0.032	[0.008–0.133]	0.001^**∗**^	0.094	[0.016–0.546]	0.008^**∗∗**^
No	139 (38.7)	220 (61.3)						
*Knowledge of Screening program*								
Yes	142 (73.2)	52 (26.8)						
No	37 (17.9)	170 (82.1)	12.546	[7.788–20.210]	0.001^**∗**^	0.861	[0.262–2.829]	0.805
*Knowledge of transmission mode*								
Yes	83 (79.8)	21 (20.2)						
No	96 (32.3)	201 (67.7)	8.275	[4.837–14.157]	0.001^**∗**^	0.687	[0.258–1.831]	0.453
*Knowledge of treatment*								
Yes	138 (51.7)	129 (48.3)						
No	41 (30.6)	93 (69.4)	2.426	[1.564–3.762]	0.001^**∗**^	0.488	[0.225–1.057]	0.069
*History of pap smear*								
Never	37 (15.7)	199 (84.3)						
More than 3 years	86 (81.9)	19 (18.1)	0.041	[0.022–0.075]	0.001^**∗**^	0.629	[0.168–2.361]	0.492
During last 3 years	56 (93.3)	4 (6.7)	0.013	[0.005–0.039]	0.001^**∗**^	0.338	[0.064–1.792]	0.202
*Gynecological examination during last 3 years*								
Yes	149 (84.2)	28 (15.8)						
No	30 (13.4)	194 (86.6)	34.412	[19.705–60.095]	0.001^**∗**^	4.517	[1.295–15.762]	0.018^**∗∗**^
*Practice of screening test*								
Yes	63 (87.5)	9 (12.5)						
No	116 (35.3)	213 (64.7)	12.836	[6.162–26.737]	0.001^**∗**^	2.430	[0.757–7.804]	0.136

^*∗*^Model included variables that were significant on univariate analyses at *p* (0.20 level).

^*∗∗*^significant at *p* value ≤ 0.05.
